# A deep learning model for real-time mortality prediction in critically ill children

**DOI:** 10.1186/s13054-019-2561-z

**Published:** 2019-08-14

**Authors:** Soo Yeon Kim, Saehoon Kim, Joongbum Cho, Young Suh Kim, In Suk Sol, Youngchul Sung, Inhyeok Cho, Minseop Park, Haerin Jang, Yoon Hee Kim, Kyung Won Kim, Myung Hyun Sohn

**Affiliations:** 1 Department of Pediatrics, Severance Children’s Hospital, Institute of Allergy, Institute for Immunology and Immunological Diseases, Brain Korea 21 PLUS Project for Medical Science, Yonsei University College of Medicine, 50-1 Yonsei-ro, Seodaemun-gu, Seoul, 03722 South Korea; 2 AITRICS, Seoul, South Korea; 3 Department of Critical Care Medicine, Samsung Medical Center, Sungkyunkwan University School of Medicine, Seoul, South Korea

**Keywords:** Machine learning, Mortality, Intensive care units, pediatric, Prognosis, Risk assessment

## Abstract

**Background:**

The rapid development in big data analytics and the data-rich environment of intensive care units together provide unprecedented opportunities for medical breakthroughs in the field of critical care. We developed and validated a machine learning-based model, the Pediatric Risk of Mortality Prediction Tool (PROMPT), for real-time prediction of all-cause mortality in pediatric intensive care units.

**Methods:**

Utilizing two separate retrospective observational cohorts, we conducted model development and validation using a machine learning algorithm with a convolutional neural network. The development cohort comprised 1445 pediatric patients with 1977 medical encounters admitted to intensive care units from January 2011 to December 2017 at Severance Hospital (Seoul, Korea). The validation cohort included 278 patients with 364 medical encounters admitted to the pediatric intensive care unit from January 2016 to November 2017 at Samsung Medical Center.

**Results:**

Using seven vital signs, along with patient age and body weight on intensive care unit admission, PROMPT achieved an area under the receiver operating characteristic curve in the range of 0.89–0.97 for mortality prediction 6 to 60 h prior to death. Our results demonstrated that PROMPT provided high sensitivity with specificity and outperformed the conventional severity scoring system, the Pediatric Index of Mortality, in predictive ability. Model performance was indistinguishable between the development and validation cohorts.

**Conclusions:**

PROMPT is a deep model-based, data-driven early warning score tool that can predict mortality in critically ill children and may be useful for the timely identification of deteriorating patients.

**Electronic supplementary material:**

The online version of this article (10.1186/s13054-019-2561-z) contains supplementary material, which is available to authorized users.

## Background

Hospitalized children, particularly those in high-acuity environments such as the pediatric intensive care unit (PICU), are inevitably susceptible to clinical deterioration. Several outcome prediction models such as the Pediatric Index of Mortality (PIM) and the Pediatric Risk of Mortality (PRISM) are widely used in PICUs [1, 2]. However, these acuity scores are based on “snapshot” values gathered during the early period following PICU admission. These static scores fail to adapt with the patient’s clinical progression and offer little assistance for the management of individual patients [3, 4].

Previous studies demonstrating that acute deterioration in patients is often preceded by subtle changes in physiological parameters [5, 6] led to the development of the Early Warning Score (EWS) [7]. Accurate and generalizable risk stratification tools may contribute to the timely identification of high-risk patients and facilitate earlier clinical intervention leading to improved patient outcomes [8]. Since its introduction, the EWS has undergone many alterations, and its modified forms are widely used in general hospitals today [9, 10]. However, the primary target population is usually confined to relatively healthy patients in general wards [9, 11] or emergency department settings [12] and may not be applicable to intensive care settings [13].

Current literature frequently calls for the development of diverse intensive care warning scores [14–16]. The rapid development in machine learning, coupled with the richness of data from extensive patient monitoring in the intensive care unit (ICU), provides unprecedented opportunities for the development of new prediction scores in the field of critical care [17–19]. Challenges in the analytics of PICU data, including pathologic diversity and complexity [20] and the wide range of age and developmental stages, are anticipated to be addressed by the implementation of innovative predictive modeling [18, 21].

Curtis et al. developed a cardiac arrest prediction model by time series trend analysis using a support vector machine algorithm that achieved excellent performance [22]. In addition, Zhengping et al. adopted Gradient Boosting Trees to learn an interpretable model, which demonstrated strong performance for the prediction of mortality and ventilator-free days in the PICU [23]. Despite their successful application of data-driven analytics, the above studies were limited by the lack of external validation. To allow practical application in a real-world setting, the preliminary results would require further refinement regarding data elements, extraction, processing, and operation with acceptable false alarms.

In this paper, we describe the development and evaluation of a new tool, the Pediatric Risk of Mortality Prediction Tool (PROMPT), for real-time mortality prediction in PICUs. We also assessed PROMPT’s suitability for practical application in the clinical care of critically ill children.

## Methods

### Study population and data sources

We used data from the electronic health records (EHRs) of all patients under 19 years old admitted to the medical ICU at Severance Hospital (Seoul, Korea) between January 2011 and December 2017. The primary cohort contained 1445 patients with 1977 ICU admissions. For external validation, we used a separate dataset provided by Samsung Medical Center (Seoul, Korea) containing data on 278 patients with 364 PICU admissions from January 2016 to November 2017. Details on these datasets are presented in Additional file 1: Table S1. All data were anonymized, and a waiver was obtained from the Institutional Review Board of each hospital (#4-2017-0060 and #2019-09015-001, respectively).

### Feature selection and data processing

The extracted data contained sets of static features, such as demographic and clinical information, and temporal features such as time-stamped vital signs. To construct a mortality prediction tool, we adopted two descriptive features—age and weight—and seven vital signs: systolic blood pressure (SBP), diastolic blood pressure (DBP), mean blood pressure (MBP), heart rate (HR), respiratory rate (RR), peripheral capillary oxygen saturation (SpO_2_), and body temperature (BT). We selected vital signs as objective predictor variables because they are routinely and frequently collected from all patients regardless of clinical situation and the values are rarely affected by the examiner. Most vital signs of ICU patients are automatically measured by monitoring devices at minimum once an hour, and the values are recorded on the EHR.

The following cleaning process ensured that the EHR data was ready for analysis and did not contain errors. Non-numeric values were removed. In addition, a set of defined ranges of physiologically possible values for selected variables were used to eliminate outliers (Additional file 1: Table S2). Carry-forward/carry-backward methods were employed for imputations. In case of multiple measurements within an hour, the most extreme values were used. Policy-based preprocessing was automated and resulted in an average coverage of 96.1% of all data with an accuracy of 97.5% compared to manual corrections. Finally, for modeling, each variable was standardized to fit an isotropic Gaussian distribution.

### Machine learning

The primary outcome was all-cause mortality in the ICU. For this binary outcome, we extracted positive instances from all cases who died in ICU and negative instances from all cases who survived (Additional file 1: Figure S1). The 24-h window of vital signs up to 6 to 60 h prior to death was extracted as a positive instance, and 24-h window of vital signs randomly chosen from during ICU stay of the survivor was assigned as a negative instance. For simplicity, only a single instance was selected from each encounter, and both sampled positive and negative instances were designated to be similar in their mean lengths to avoid possible biases (Additional file 1: Table S3).

Model development was carried out using convolutional neural networks (CNNs) [24], a class of deep, feed-forward artificial neural networks consisting of alternating convolutional and subsampling layers that replicate the complexities of the animal visual cortex. The convolution operation involves combining input data with a convolution kernel to form transformed data. The filters in the convolutional layers are modified based on learned parameters to incorporate the most useful information for a specific task. This method adjusts automatically to determine the best feature based on the task and has achieved great success in feature representation learning in images [25]. Recent reports have also demonstrated its utility in predicting sepsis in adult [26] and pediatric populations [27] and cardiac arrhythmias [28]. A detailed architecture of our CNN, which consisted of two layers of one-dimensional convolutional operations followed by max pooling is presented in the supplementary materials for reproducibility (Additional file 1: Table S4). A fivefold cross-validation with five repetitions on the development cohort was adopted to validate PROMPT’s performance, and external validation was followed to assess its generalizability.

### Statistical analysis

We compared the performance of PROMPT with other standard machine learning algorithms, such as Gradient Boosting Decision Trees (GBDT) [29], Long Short-Term Memory (LSTM) [30], and the Pediatric Index of Mortality 3 (PIM 3), which is currently widely used in PICUs [1]. Model performance was assessed based on discrimination using the area under the receiver operating characteristic curve (AUROC), one of the most commonly used metrics, and the area under the precision-recall curve (AUPRC), which, because the outcome of interest was mortality, was calculated considering a skewed large domain of true negatives [31]. Sensitivity, specificity, positive predicted value (PPV), negative predicted value (NPV), and accuracy were also evaluated for all prediction tools assessed in this study.

## Results

### Dataset statistics

As shown from the descriptive statistics for each cohort (Additional file 1: Table S1), the development cohort consisted of 1977 patient encounters, in which 303 cases of mortality (15.3%) were identified. The validation cohort showed 9.6% mortality. Significant differences were noted between the two datasets in terms of age, PIM 3, mortality, length of ICU stay, and inclusion period.

### Mortality prediction performance

The performance metrics of PROMPT on mortality prediction compared to other standard machine learning algorithms and PIM 3 are summarized in Table 1. The best performance was achieved for predicting mortality 6 h prior to death (AUROC 0.965, AUPRC 0.831) with a slight decrease, although still high-performance, as the time window increased to 60 h prior to death. In detecting mortality 60 h in advance, PROMPT (AUROC 0.887, AUPRC 0.565) consistently outperformed GBDT (AUROC 0.831, AUPRC 0.419), LSTM (AUROC 0.814, AUPRC 0.429), and PIM 3 (AUROC 0.785, AUPRC 0.298) in the development cohort (Table 1), also shown in the micro-averaged performance comparisons (Additional file 1: Figure S2). Similar results were found on external validation (Table 1).

**Table 1:** Summary of model mortality detection performance

	Development cohort				Validation cohort			
Lead time window	AUROC	95% CI	AUPRC	95% CI	AUROC	95% CI	AUPRC	95% CI
PROMPT								
6 h	0.965	± 0.006	0.831	± 0.018	0.922	± 0.004	0.716	± 0.016
12 h	0.948	± 0.009	0.745	± 0.029	0.945	± 0.004	0.701	± 0.023
24 h	0.933	± 0.009	0.733	± 0.027	0.946	± 0.005	0.605	± 0.024
48 h	0.899	± 0.013	0.570	± 0.041	0.849	± 0.007	0.360	± 0.023
60 h	0.887	± 0.018	0.565	± 0.052	0.881	± 0.011	0.445	± 0.031
GBDT								
6 h	0.944	± 0.008	0.767	± 0.022	0.877	± 0.005	0.499	± 0.032
12 h	0.927	± 0.008	0.684	± 0.028	0.915	± 0.005	0.605	± 0.022
24 h	0.908	± 0.014	0.612	± 0.032	0.897	± 0.007	0.442	± 0.021
48 h	0.853	± 0.014	0.452	± 0.031	0.805	± 0.009	0.342	± 0.025
60 h	0.831	± 0.022	0.419	± 0.051	0.790	± 0.012	0.403	± 0.035
LSTM								
6 h	0.945	± 0.010	0.808	± 0.019	0.875	± 0.006	0.547	± 0.039
12 h	0.915	± 0.016	0.703	± 0.031	0.870	± 0.012	0.520	± 0.034
24 h	0.889	± 0.013	0.644	± 0.032	0.837	± 0.012	0.348	± 0.032
48 h	0.844	± 0.014	0.530	± 0.029	0.770	± 0.013	0.348	± 0.027
60 h	0.814	± 0.025	0.429	± 0.050	0.759	± 0.019	0.353	± 0.034
PIM 3								
Total	0.767	–	0.509	–	0.881	–	0.500	–
Subset 1^*^	0.787	–	0.315	–	0.876	–	0.462	–
Subset 2^**^	0.785	–	0.298	–	0.876	–	0.462	–

*AUROC* area under the receiver operating characteristic curve, *CI* confidence interval, *AUPRC* area under the precision-recall curve, *PROMPT* pediatric risk of mortality prediction tool, *GBDT* Gradient Boosting Decision Trees, *LSTM* Long Short-Term Memory, *PIM 3* Pediatric Index of Mortality 3

^*^Subset of the cohort with data of at least 48 h

^**^Subset of the cohort with data of at least 60 h

Additional paired comparison metrics at a sensitivity of 0.8 for specificity, PPV, NPV, and accuracy for each model are presented in Table 2. Within the development cohort, PROMPT identified 80% of patients who were to die in 24 h, yielded 7% false alarms (specificity = 0.931), and was the most consistently accurate of all metrics. Comparison of sensitivity according to the number of false alarms showed that PROMPT provided fewer false alarms than existing models, including PIM 3, in both cohorts (Additional file 1: Figure S3).

**Table 2:** Comparison of model’s accuracy for mortality prediction

	Development cohort					Validation cohort				
Lead time window	Sensitivity	Specificity	PPV	NPV	Accuracy	Sensitivity	Specificity	PPV	NPV	Accuracy
PROMPT										
6 h	0.846	0.963	0.663	0.986	0.953	0.800	0.850	0.288	0.982	0.846
12 h	0.800	0.946	0.555	0.983	0.935	0.800	0.890	0.336	0.985	0.884
24 h	0.800	0.931	0.454	0.985	0.922	0.849	0.887	0.334	0.989	0.884
48 h	0.800	0.834	0.224	0.986	0.832	0.800	0.752	0.177	0.983	0.755
60 h	0.800	0.882	0.268	0.988	0.878	0.800	0.772	0.190	0.983	0.773
GBDT										
6 h	0.800	0.933	0.509	0.982	0.922	0.800	0.805	0.238	0.981	0.805
12 h	0.801	0.898	0.398	0.982	0.891	0.800	0.854	0.276	0.984	0.850
24 h	0.800	0.854	0.283	0.983	0.850	0.800	0.818	0.227	0.984	0.817
48 h	0.800	0.769	0.172	0.985	0.771	0.800	0.629	0.126	0.979	0.640
60 h	0.800	0.693	0.123	0.985	0.698	0.800	0.551	0.107	0.976	0.567
LSTM										
6 h	0.800	0.951	0.588	0.982	0.939	0.800	0.770	0.209	0.981	0.772
12 h	0.800	0.888	0.374	0.981	0.881	0.800	0.782	0.204	0.982	0.783
24 h	0.800	0.828	0.251	0.983	0.826	0.800	0.740	0.170	0.982	0.743
48 h	0.800	0.729	0.150	0.984	0.733	0.800	0.537	0.104	0.976	0.554
60 h	0.800	0.626	0.103	0.983	0.635	0.800	0.505	0.098	0.974	0.524
PIM 3										
Total	0.800	0.617	0.392	0.909	0.661	0.800	0.799	0.298	0.974	0.799
Subset 1^*^	0.806	0.643	0.218	0.964	0.661	0.818	0.754	0.182	0.984	0.758
Subset 2^**^	0.800	0.643	0.200	0.966	0.659	0.818	0.754	0.182	0.984	0.758

*PPV* positive predictive value, *NPV* negative predictive value, *PROMPT* pediatric risk of mortality prediction tool, *GBDT* Gradient Boosting Decision Trees, *LSTM* Long Short-Term Memory, *PIM 3* Pediatric Index of Mortality 3

^*^Subset of the cohort with data of at least 48 h

^**^Subset of the cohort with data of at least 60 h

### Visualization of prediction trajectory

**Fig. 1:**
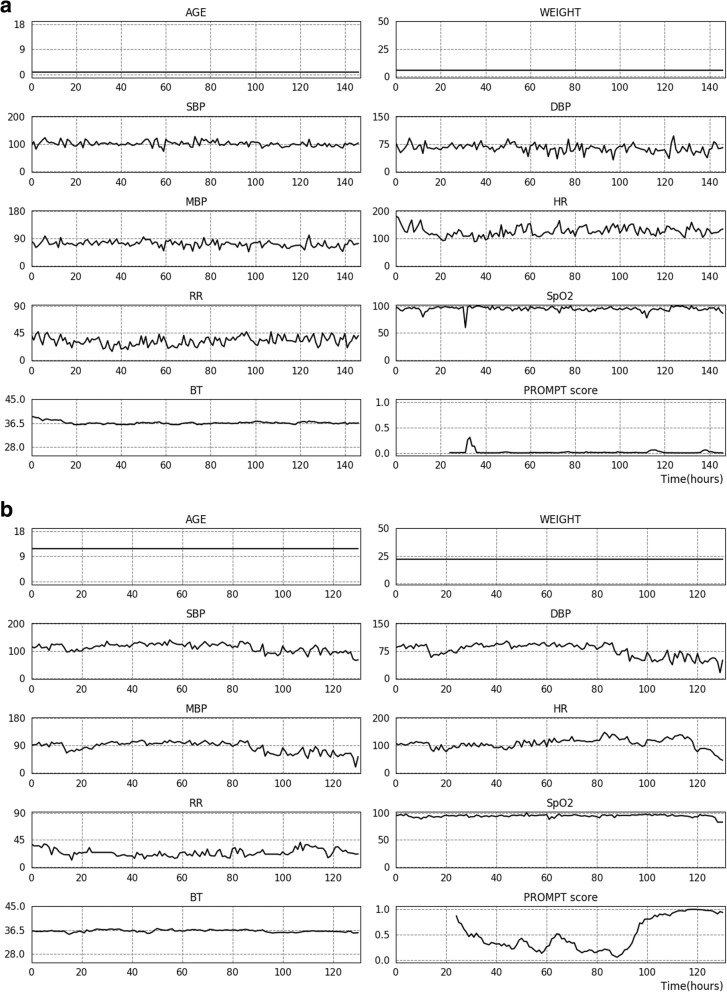
Prediction trajectory using PROMPT. Serial trends of recorded vital signs during ICU stay and hourly calculated predicted mortality rate using PROMPT shown for patients from the validation cohort who survived (**a**) and died (**b**), respectively. Predicted mortality was averaged over multiple prediction models trained from development cohort using the dataset to predict mortality in the preceding *k* hours (where *k* = 6, 12, 24, 48, and 60). SBP, systolic blood pressure; DBP, diastolic blood pressure; MBP, mean blood pressure; HR, heart rate; RR, respiratory rate; SpO_2_, peripheral capillary oxygen saturation; BT, body temperature; ICU, intensive care unit

PROMPT produced an averaged mortality risk score over multiple prediction models trained from development cohorts to predict mortality in the preceding 6, 12, 24, 48, and 60 h (examples are presented in Fig. 1). Where *t* is the current time point, the input data composed of two descriptive features and vital signs in a range of [*t* − 24, *t*] transformed to an averaged risk score. The same procedure was repeated at the *t* + 1 time points to generate prediction trajectory. A sliding window (0 to 24 h) moved hour-by-hour through the time series to generate predicted mortality for each time point during the ICU stay.

### Designation of time and feature contributions

**Fig. 2:**
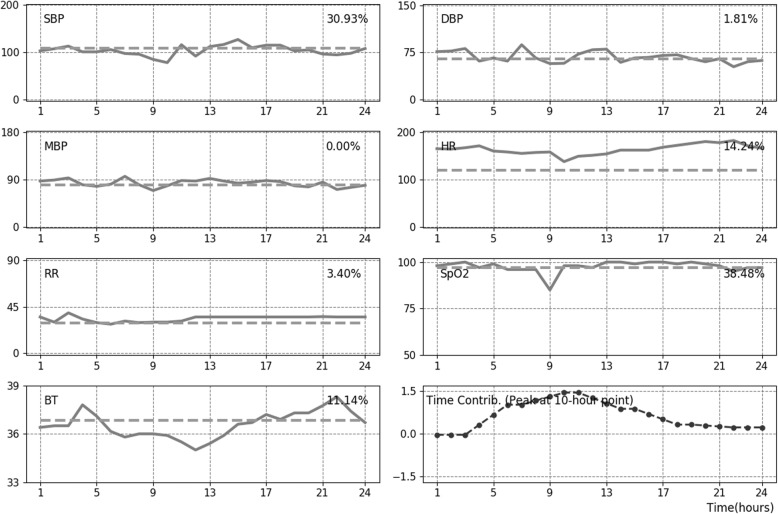
Depiction of time and feature contributions for mortality using PROMPT. Measured contribution (%) for mortality at the critical time point and serial trend of vital signs over 24 h are plotted on each panel. The last sub-figure presents the time contribution. The height of the graph represents the level of importance, and the positive/negative conversion distinguishes the time point contributed to make positive or negative predictions for mortality. In the presented case, the critical time point (i.e., a peak of time contribution) was about 10 h, of which fluctuations in SpO_2_, blood pressure, and HR are shown to contribute to instability which can be associated with mortality. SBP, systolic blood pressure; DBP, diastolic blood pressure; MBP, mean blood pressure; HR, heart rate; RR, respiratory rate; SpO_2_, peripheral capillary oxygen saturation; BT, body temperature

An interpretation module that quantitatively measured the contribution of time series features for mortality was developed. Every time-stamped vital sign was substituted for an age-dependent mean value and, following changes in predicted mortality, produced quantitative contributions of each feature (%). In addition, algebraic manipulation demonstrated that the contribution of each time point was captured by the importance of six blocks in making a prediction. This is because a temporal relationship is lost due to pooling operations. Accordingly, 24 h data was grouped by six blocks for which the contribution to the prediction was computed. An average filter was then applied for smoothing the signal. Figure 2 depicts illustrative examples of a deceased case showing the measured contribution of each time point and those of features at the most critical time point, as well as the linear trend of vital signs for 24 h.

### Interpretability of PROMPT

Individual feature importance was plotted by computing a sensitivity heat map on a total of 363 test instances in the development cohort to measure the importance of individual input variables (Additional file 1: Figure S4). The sensitivity was defined as the derivative of the predicted mortality according to the input variables, and the relative importance of input variables was normalized to satisfy the sum-to-one constraint. RR showed the highest relative importance among all other features, followed by SBP and HR.

Individual Conditional Expectation plots are shown in Additional file 1: Figure S5. These six plots show the test instances in the development cohort. The predicted mortality probability was computed by creating variants of each input variable while keeping all other features as it is. For blood pressures, predicted mortality tends to increase when pressure values are too low or too high, and it declines as the feature value falls within the physiological range. A similar trend was observed in other vital signs, such as HR, RR, and BT. In SpO_2_, predicted mortality tends to decrease as the degree of saturation increases to 100%. However, several instances were identified with a high mortality probability despite a high SpO_2_, due to the correlation between the features.

## Discussion

In this study, we developed and validated a targeted real-time early warning score, PROMPT, based on a CNN algorithm using a PICU dataset with routine vital signs. Utilizing a handful of variables, PROMPT achieved high performance with high sensitivity and specificity for predicting mortality in PICU patients. In predictive ability, it outperformed the conventional severity scoring system, PIM 3, as well as other models that use GBDT and LSTM.

Existing risk prediction tools in ICU use static physiological parameters from early in the course of critical illness (often within the first 24 h following admission), along with other components, such as age and diagnosis, to assess severity and risk of death for the purpose of predicting outcomes [32]. For pediatric populations, PIM and PRISM are the most representative [1, 2]. However, it is generally agreed that they are poor surrogates for risk stratification and should not be used as the basis for individual treatment decisions [4, 33, 34]. Generic severity scores were originally developed and calibrated to maximize the capacity for mortality risk assessment for populations of interest, and not for clinical decision-making concerning individuals within those populations [4]. Moreover, utilizing the poorest values within a fixed time window, regardless of the outcome of interventions, fails to reflect the dynamic clinical course including differential treatment responses. Thus, these systems are unable to distinguish which patients are at higher risk of developing specific acute conditions. In our study, this was demonstrated by the notably low discriminative ability of PIM 3 in mortality prediction.

Predictive analytics on time series monitoring data were introduced [35, 36] based on evidence that physiologic signatures preceded acute deterioration of patients prior to the arousal of clinical suspicion [5, 6, 37]. Widespread adoption of EHRs which could be queried in real time enabled the development of EWS with the ability to identify clinically deteriorating patients in need of intervention [8, 38]. Accordingly, a wide variety of different tools now exist and are operated alongside rapid response teams in different hospital contexts [9, 10, 39]. For instance, the Bedside Pediatric Early Warning Score (PEWS) is used across the UK National Health Service for the detection of patients in wards who are at risk of acute deterioration, facilitating their timely upgrade to higher level care [40, 41]. Similarly, many other EWS systems have been developed and validated primarily on general wards [11, 40], and their use has been extended to emergency departments [12, 42] and prehospital settings [43].

The ICU environment, where patients are clinically unstable and change rapidly between states of improvement and deterioration, calls for meticulous monitoring and clinical support. This has facilitated the development of ICU early warning systems [18, 44, 45]. The development of more sophisticated monitoring devices has resulted in an exponential growth in sensor data. This, coupled with recent advances in machine learning, artificial intelligence techniques, and data archiving hardware, has facilitated the discovery of data-driven characteristics and patterns of diseases [18, 36, 46–48]. However, the numerous developmental stages, baseline age-related differences in physiologic parameters, and the wide range of underlying pathologic diversity present unique challenges for the analysis of PICU patient data [20, 21]. Moreover, physiological data of the patient is continuously influenced by clinical interventions such as oxygen supplement, volume resuscitation, and vasopressor use, given that the core principle of intensive care is to maintain the steady state [20]. Because variations in physiological data occur within a complex biological system composed of multiple components that interact together, more sophisticated deep learning models such as neural networks, which automatically learn features, have demonstrated better performance than traditional machine learning [49].

Our study makes several significant contributions to the existing literature on mortality prediction in the PICU setting. PROMPT utilized changing vital signs of individuals; employed CNN, a deep model primarily used in image analytics; and achieved high accuracy and discriminative ability in predicting mortality. Prediction performance decreased slightly as the time window ahead of the event lengthened from 6 to 60 h, and the performance of this earlier identification was relatively lower in the validation cohort. Nevertheless, PROMPT provided AUROC above 0.88 for predicting mortality 60 h in advance from both cohorts. Moreover, it consistently achieved higher sensitivity and specificity compared to other standard machine learning algorithms and PIM 3.

Accuracy and false alarm rate are important issues to consider in the practical implementation of EWS in ICU settings. Because sensitivity and specificity mutually interact, the performance of EWS and alarm fatigue should be weighed and optimized [50]. Notably, PROMPT consistently provided higher specificity than PIM 3 and other algorithms against which it was tested. In addition, PROMPT maintained a higher level of accuracy than other models even with a small number of alarms (Additional file 1: Figure S3).

In this study, PROMPT used seven vital signs along with the patient’s age and body weight on PICU admission. The model does not require any custom data entry and relies entirely on data elements that are usually available from the EHRs of most hospitals. Incorporating further parameters such as laboratory tests would be expected to enhance PROMPT’s performance. However, we note that models based on continuously updated physiologic monitoring data are better able to provide timely warning of pending deterioration. Thus, using only the most basic and commonly measured critical care data streamed from the bedside monitor has an advantage for the broader adoption of this model in other ICUs. Relatively minimal data requirements, few manual data entry requirements, and automated operation on data extracted from EHRs save additional labor and cost and may lighten the burden of application in the clinical setting.

This study has several limitations. First, we could not determine the generalizability of our results to other populations. In addition, the retrospective study design did not allow the determination of model performance in a prospective setting. Our model remains a population-based estimate, as we did not validate its efficacy for individual prognostication in a prospective way. Moreover, despite PROMPT’s high performance in detecting and predicting mortality, this knowledge alone is insufficient to affect patient outcomes. Clinician input is required to determine clinical interventions and shape patient-centered outcomes.

However, considering that clinicians in the PICU environment face limited clinical resources and that rationing of health care is a reality in some respects, PROMPT may have the potential to benefit clinical practice. If the risk of critical adverse outcomes is identified earlier, clinicians could allocate staffing and other medical resources with a higher level of certainty. Our model utilizes easily collected data and, therefore, may be particularly suitable for bedside prognostications in relatively low-resourced environments.

In addition, because the predictive window of PROMPT is up to 60 h before death, earlier warnings may give physicians more time to intervene and prevent or mitigate mortality. Alternatively, once physicians are alerted and prepared for the likelihood of death, there are opportunities for preference-concordant, high-value care in PICUs by initiating goals of care discussions earlier and revising treatment plans. Hence, our future work will focus on the practical impact of early recognition of at-risk patients on clinically relevant outcomes.

Lastly, we would like to stress the additional implications of our model. Although our current model does not tell the clinician precisely how to treat a deteriorating patient, the trajectory of predicted risk and designation for time and feature contributions are expected to provide additional information, indirectly. Changes in the trend of predicted mortality over time, coupled with an event or specific intervention with a patient, may provide clinicians intuitive insight into potential associations with a favorable or unfavorable clinical course in individual cases.

## Conclusion

In this two-center retrospective study, we validated an easily implementable deep model-based real-time mortality prediction system for critically ill children. Using seven vital signs routinely recorded in standard critical care practice, along with patient age and body weight on ICU admission, our results indicate that PROMPT provides high sensitivity, specificity, and discriminative ability for the prediction of patients at high risk for mortality up to 60 h prior to death. This data-driven early warning score may be an effective tool for the timely recognition of deteriorating patients.

## Additional file

Additional file 1: Supplementary figures and tables. (DOCX 980 kb)

## Data Availability

The data that support the findings of this study are available on request from the corresponding author. The data are not publicly available because they contain information that could compromise research participant privacy.

## Abbreviations

AUPRC: Area under the precision-recall curve
AUROC: Area under the receiver operating characteristic curve
BT: Body temperature
CNN: Convolutional Neural Network
DBP: Diastolic blood pressure
EHR: Electronic health records
EWS: Early Warning Score
GBDT: Gradient Boosting Decision Trees
HR: Heart rate
ICU: Intensive care unit
LSTM: Long Short-Term Memory
MBP: Mean blood pressure
NPV: Negative predicted value
PICU: Pediatric intensive care unit
PIM 3: Pediatric Index of Mortality 3
PPV: Positive predicted value
PRISM: Pediatric Risk of Mortality
PROMPT: Pediatric Risk of Mortality Prediction Tool
RR: Respiratory rate
SBP: Systolic blood pressure
SpO_2_: Peripheral capillary oxygen saturation
