# Supplementary material for: A deep learning model for real-time mortality prediction in critically ill children
Source: Crit Care. 2019 Aug 14;23:279. doi: 10.1186/s13054-019-2561-z (PMC6694497; doi:10.1186/s13054-019-2561-z)
Supplement: Supplementary file 1 — Supplementary figures and tables. (DOCX 980 kb) [file 13054_2019_2561_MOESM1_ESM.docx]

**SUPPLEMENTAL DIGITAL CONTENT**

**Figure S1. Conceptual illustration of extracting positive and negative instances from each encounter**

(a) Extracting positive instance from mortality case


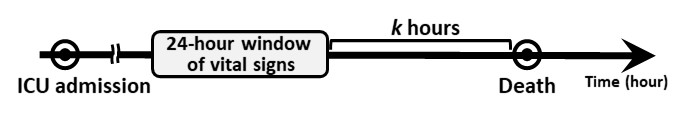


(b) Extracting negative instance from survival case


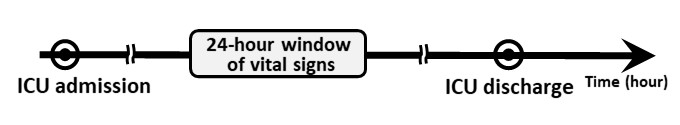


24-hour window of vital signs in *k* hour (where *k* = 6, 12, 24, 48, or 60) prior to time of death was extracted as a positive instance. 24-hour window of vital signs randomly selected from during ICU stay of the survivor was designated as a negative instance. For simplicity, only a single instance was chosen from each encounter.

ICU, intensive care unit

**Figure S2-1. Receiver-operating characteristic curve and precision-recall curve for predicting all-cause mortality: 24 hours in advance**

(a)


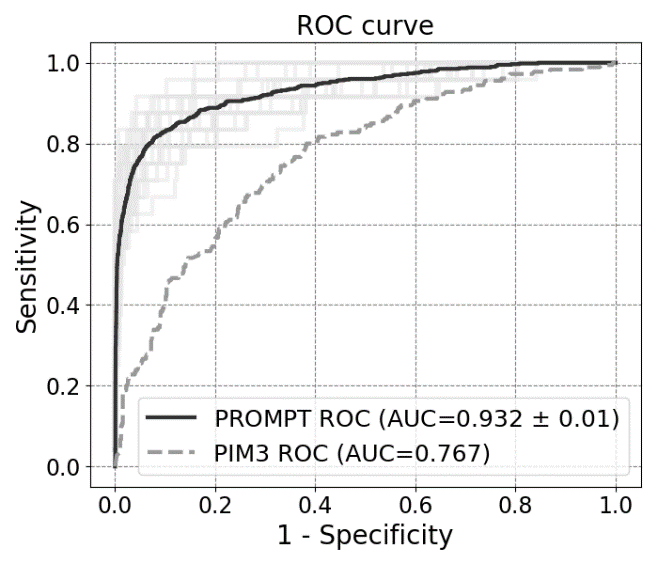

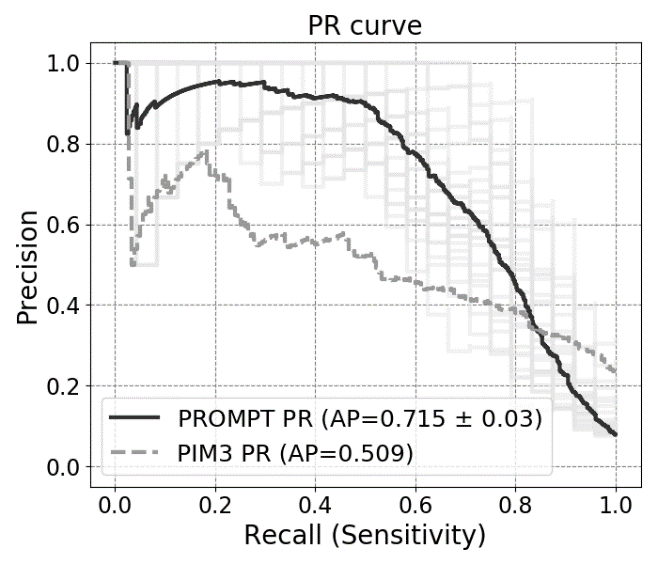


(b)


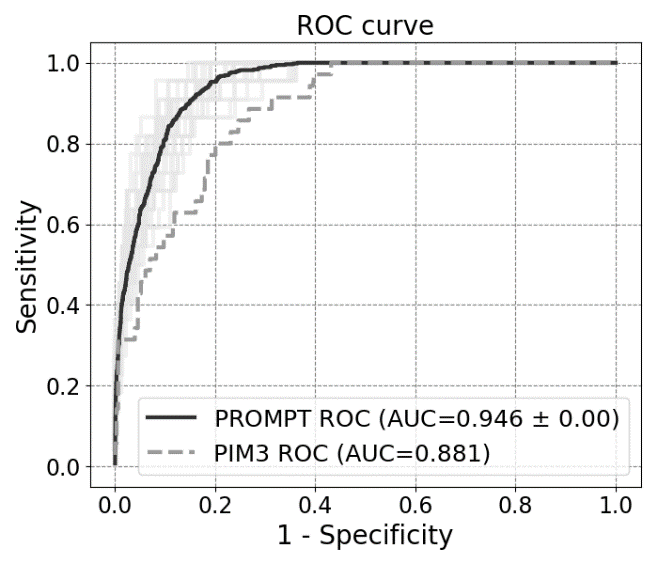

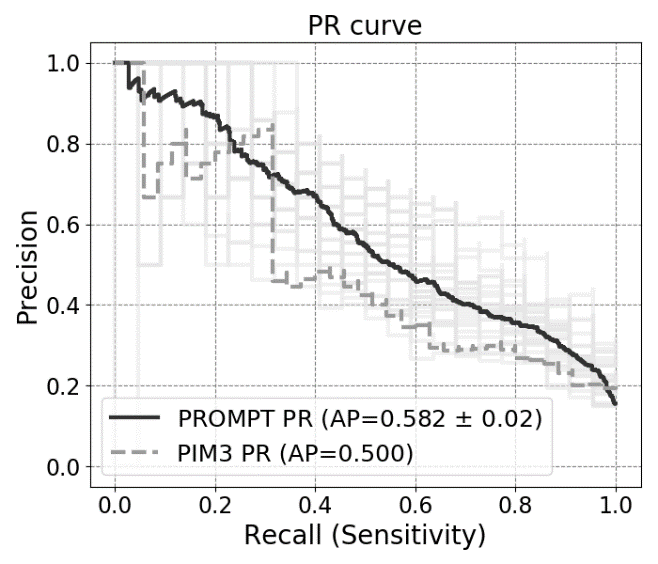


Area under the receiver-operating characteristic curve (AUROC) and area under the precision-recall curve (AUPRC) trained on each cohort for mortality prediction up to 24 hours prior to death. Results of 5-fold cross-validations on (a) development cohort and (b) validation cohort. Dark solid line represents micro-averaged ROC and PR curves for PROMPT from 5-fold cross-validations with five repetitions. Light solid lines represent each validation. Dotted line shows ROC and PR curves for PIM 3.

PROMPT, Pediatric Risk of Mortality Prediction Tool; PIM 3, Pediatric Index of Mortality 3

**Figure S2-2. Receiver-operating characteristic curve and precision-recall curve for predicting all-cause mortality: 60 hours in advance**

(a)


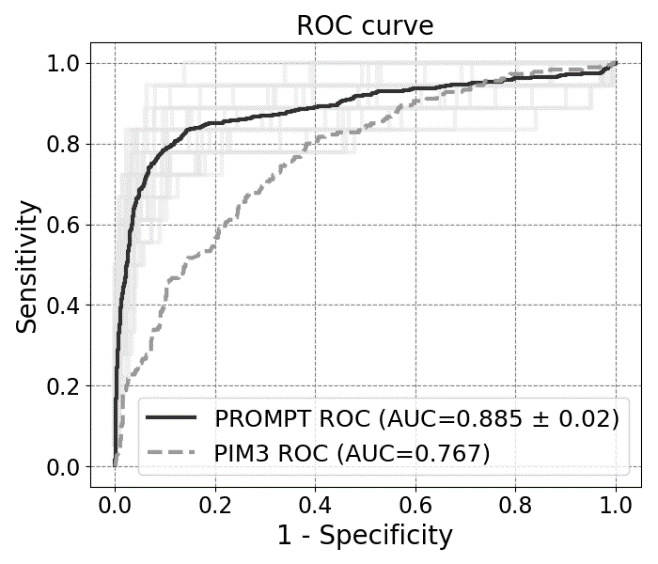

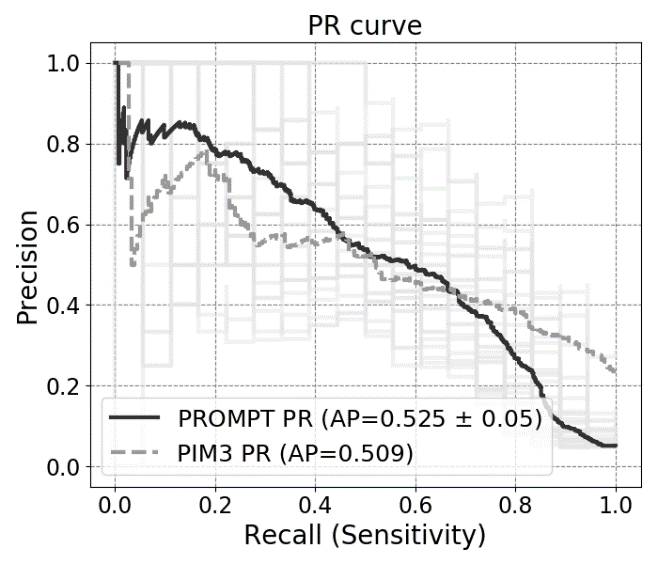


(b)


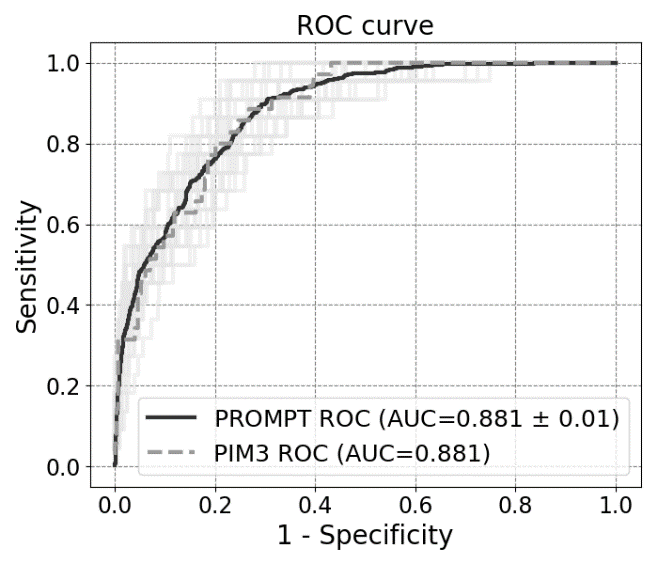

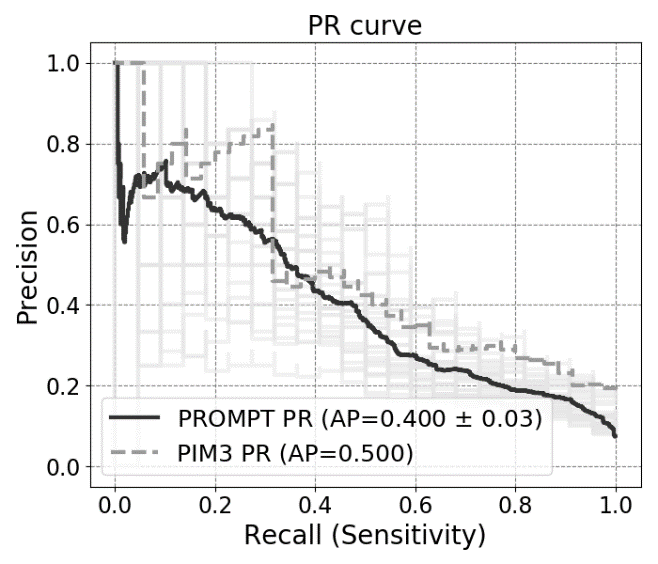


Area under the receiver-operating characteristic curve (AUROC) and area under the precision-recall curve (AUPRC) trained on each cohort for mortality prediction up to 60 hours prior to death. Results of 5-fold cross-validations on (a) development cohort and (b) validation cohort. Dark solid line represents micro-averaged ROC and PR curves for PROMPT from 5-fold cross-validations with five repetitions. Light solid lines represent each validation. Dotted line shows ROC and PR curves for PIM 3.

PROMPT, Pediatric Risk of Mortality Prediction Tool; PIM 3, Pediatric Index of Mortality 3

**Figure S3. Sensitivity for number of alarms**

(a) (b)

**
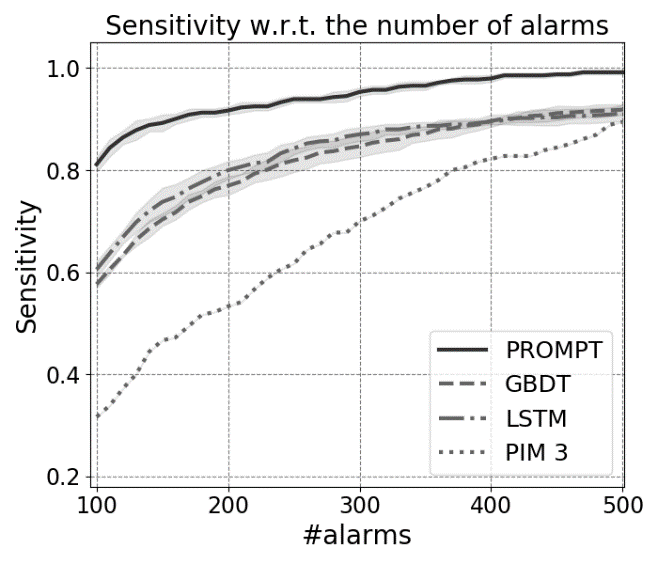

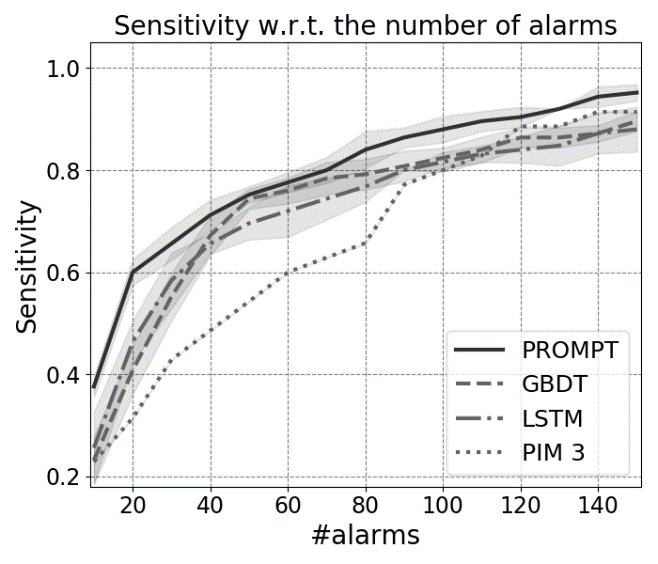
**

Comparison of sensitivity by the number of alarms for PROMPT, other standard machine learning algorithms, and PIM 3. All models were trained from data up to 6 hours prior to death from the development cohort (a) and the validation cohort (b). Solid and dotted lines show the mean from five-fold cross-validations with five repetitions. Shaded regions represent standard deviation.

**Figure S4. Relative feature importance**

**
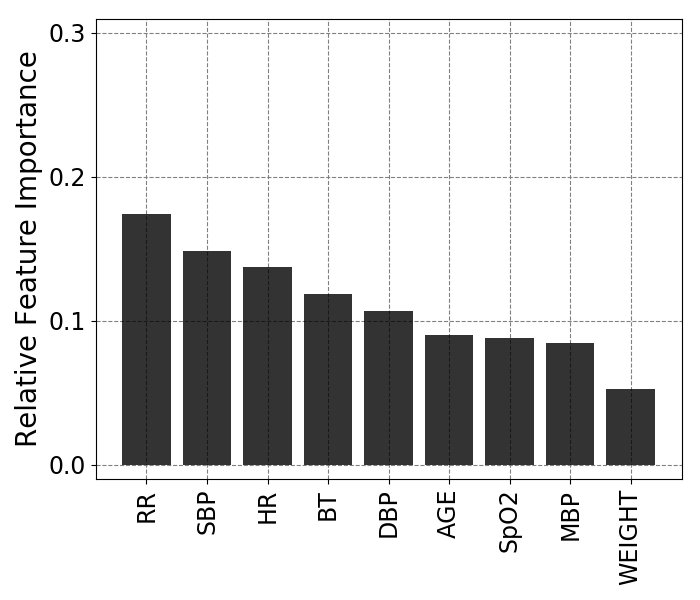
**

The bars represent the relative importance of input variables, which the sum of values equal one.

RR, respiratory rate; SBP, systolic blood pressure; HR, heart rate; BT, body temperature; DBP, diastolic blood pressure; SpO_2_, peripheral capillary oxygen saturation; MBP, mean blood pressure

**Figure S5. Individual Conditional Expectation plots of mortality probability for each vital sign**

| 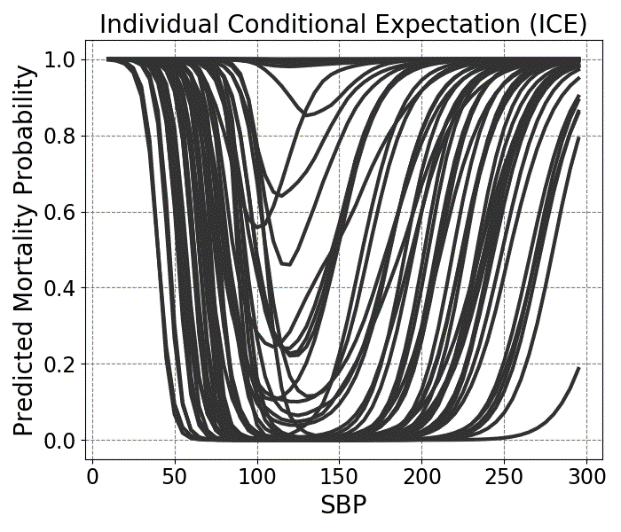 | 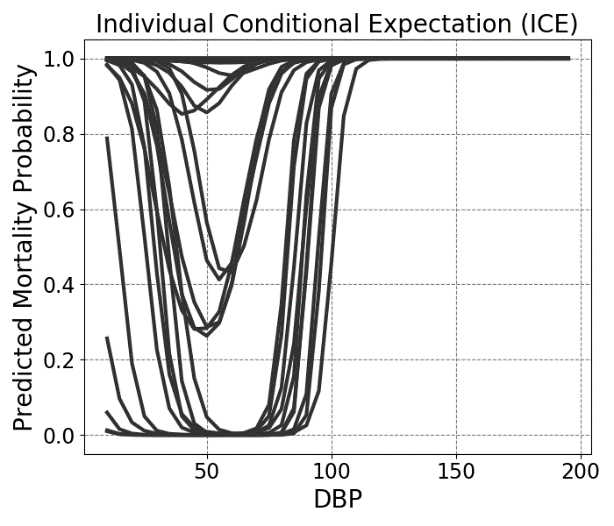 |
| --- | --- |
| 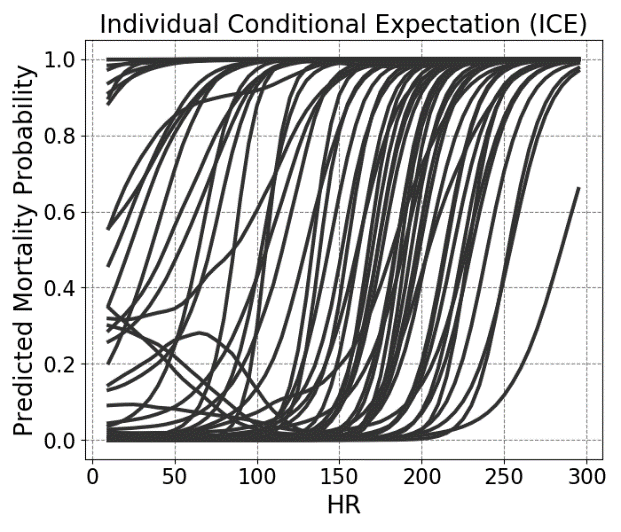 | 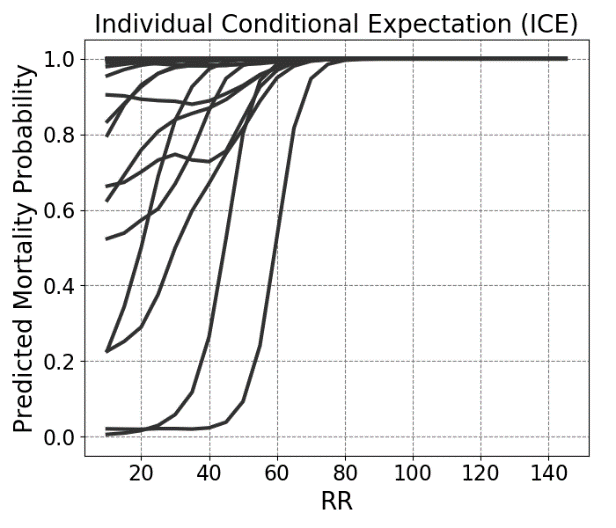 |
| 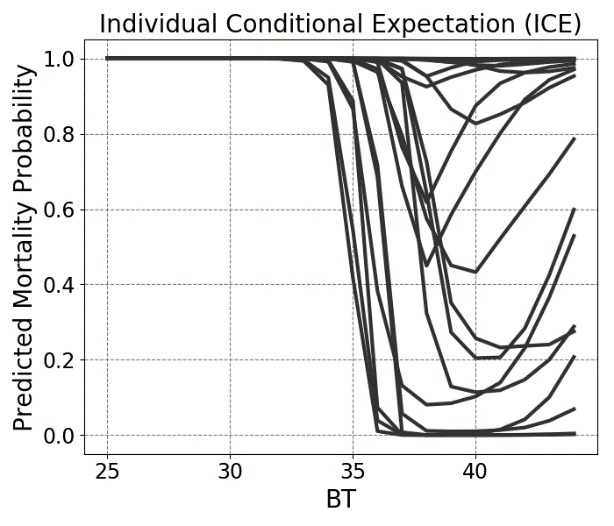 | 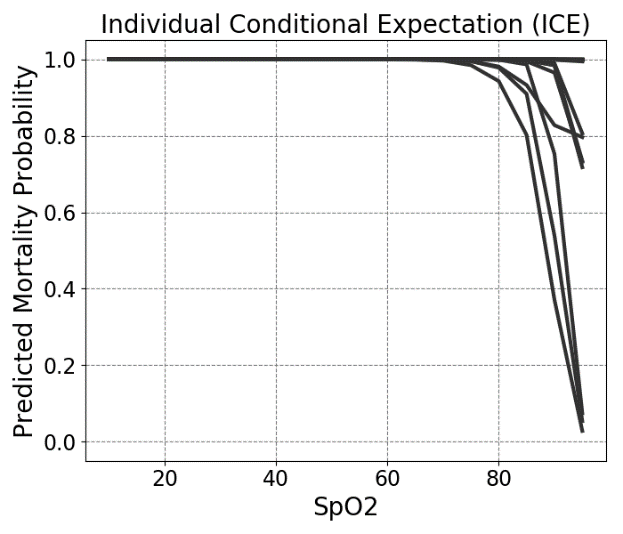 |

Individual Conditional Expectation plots of predicted mortality probability for each vital sign (SBP, DBP, HR, RR, BT, and SpO_2_) from the test instances of the development cohort. The dark solid line represents an individual instance with the value of each vital sign and the corresponding predictions.

SBP, systolic blood pressure; DBP, diastolic blood pressure; HR, heart rate; RR respiratory rate; BT, body temperature; SpO_2_, peripheral capillary oxygen saturation

**Table S1. Demographic and clinical characteristics of each dataset**

| **Variables** | **Development cohort**  **(Severance Hospital)** | **Validation cohort**  **(Samsung Medical Center)** | ***P*-value** |
| --- | --- | --- | --- |
| Study period | Jan 2011 – Dec 2017 | Jan 2016 – Nov 2017 |  |
| Hospital character | University-affiliated hospital | University-affiliated hospital |  |
| Patients, n | 1,445 | 278 |  |
| Patient encounters, n | 1,977 | 364 |  |
| Age, years | 5.9 ± 5.8 | 4.3 ± 5.0 | < 0.001 |
| Male sex | 1,096 (55.4) | 196 (53.8) | 0.576 |
| PIM 3 | 0.161 ± 0.257 | 0.116 ± 0.227 | 0.003 |
| Deaths | 303 (15.3) | 35 (9.6) | 0.001 |
| Length of stay, days | 8.6 ± 21.6 | 12.8 ± 28.4 | 0.007 |

PIM 3, Pediatric Index of Mortality 3

Data are expressed as n (%) or mean $\pm$ SD

**Table S2. Summary of defined ranges of selected variables**

| **Variables** | **Minimum** | **Maximum** |
| --- | --- | --- |
| SBP | 0 | 300 |
| DBP | 0 | 200 |
| MBP | 0 | 250 |
| HR | 0 | 300 |
| RR | 0 | 150 |
| SpO_2_ | 0 | 100 |
| BT | 25 | 45 |

SBP, systolic blood pressure; DBP, diastolic blood pressure; MBP, mean blood pressure; HR, heart rate; RR, respiratory rate; SpO_2_, peripheral capillary oxygen saturation; BT, body temperature

**Table S3. Brief statistics of generated positive and negative instances**

|  | | Number of cases | Positive rate (%) | Length of positive instances (mean ± SD) | Length of negative instances (mean ± SD) |
| --- | --- | --- | --- | --- | --- |
| **Development cohort** | 6 hrs | 1,821 | 8.1 | 21.35 ± 5.63 | 21.40 ± 5.56 |
|  | 12 hrs | 1,814 | 7.7 | 20.96 ± 6.10 | 21.09 ± 5.94 |
|  | 24 hrs | 1,794 | 6.7 | 21.48 ± 5.96 | 21.22 ± 6.02 |
|  | 48 hrs | 1,777 | 5.8 | 22.03 ± 5.68 | 21.50 ± 5.90 |
|  | 60 hrs | 1,767 | 5.3 | 23.27 ± 3.27 | 22.33 ± 4.22 |
| **Validation cohort** | 6 hrs | 354 | 7.1 | 20.84 ± 7.41 | 20.88 ± 7.37 |
|  | 12 hrs | 352 | 6.5 | 23.17 ± 3.87 | 22.89 ± 4.09 |
|  | 24 hrs | 351 | 6.3 | 24.00 ± 0.00 | 23.50 ± 1.88 |
|  | 48 hrs | 351 | 6.3 | 24.00 ± 0.00 | 23.50 ± 1.88 |
|  | 60 hrs | 351 | 6.3 | 24.00 ± 0.00 | 23.50 ± 1.88 |

**Table S4. Network architecture of PROMT**

| **Layer name** | Components | |
| --- | --- | --- |
| **Conv1** | 1x1 conv., #channels=256, strides=1  for static variables (age, weight) | 1x5 conv., #channels=256, strides=1  for dynamic variables (vital signs) |
|  | Concatenation along an axis for filters  Batch normalization  ReLu | |
| **Pool1** | 1x5 max pooling. strides=2  Dropout (0.5) | |
| **Conv2** | 1x5 conv., #channels=512, strides=1  Batch normalization  ReLu | |
| **Pool2** | 1x5 max pooling. strides=2  Dropout (0.5) | |
| **FC (logit)** | Fully-connected layers followed by a sigmoid function | |
